# Measuring hemoglobin spectra: searching for carbamino-hemoglobin

**DOI:** 10.1117/1.JBO.25.10.105001

**Published:** 2020-10-23

**Authors:** Emmanuel Dervieux, Quentin Bodinier, Wilfried Uhring, Michaël Théron

**Affiliations:** a BiOSENCY, Cesson-Sévigné, France; b University of Strasbourg and CNRS, Strasbourg Cedex, France; c Université de Bretagne Occidentale, ORPHY, Brest, France

**Keywords:** spectrophotometry, hemoglobin, pulse oximetry, transcutaneous monitoring, photoplethysmography

## Abstract

**Significance:** The arterial carbon dioxide (${\mathrm{CO}}_{2}$) partial pressure ${\mathrm{Pa}}_{{\mathrm{CO}}_{2}}$ is a clinically relevant variable. However, its measurement requires arterial blood sampling or bulky and expensive transcutaneous ${\mathrm{Ptc}}_{{\mathrm{CO}}_{2}}$ meters. While the spectrophotometric determination of hemoglobin species—such as oxy-hemoglobin ($O_{2}\mathrm{Hb}$) and deoxy-hemoglobin (HHb)—allowed for the development of pulse oximetry, the measurement of ${\mathrm{CO}}_{2}$ blood content with minimal discomfort has not been addressed yet.

**Aim:** Characterizing human carbamino-hemoglobin (${\mathrm{CO}}_{2}\mathrm{Hb}$) absorption spectrum, which is missing from the literature. Providing the theoretical background that will allow for transcutaneous, noninvasive ${\mathrm{Pa}}_{{\mathrm{CO}}_{2}}$ measurements.

**Approach:** A tonometry-based approach was used to obtain gas-equilibrated, lysed, diluted human blood. Equilibration was performed with both ${\mathrm{CO}}_{2}$, dinitrogen ($N_{2}$), and ambient air. Spectrophotometric measurements were carried out on the 235- to 1000-nm range. A theoretical background was also derived from that of pulse oximetry.

**Results:** The absorption spectra of both ${\mathrm{CO}}_{2}\mathrm{Hb}$ and HHb were extremely close and comparable with that of state-of-the-art HHb. The above-mentioned theoretical background led to an estimated relative error above 30% on the measured amount of ${\mathrm{CO}}_{2}\mathrm{Hb}$ in a subject’s blood. Auxiliary measurements revealed that the use of ethylene diamine tetraacetic acid did not interfere with spectrophotometric measurements, whereas sodium metabisulfite did.

**Conclusions:** ${\mathrm{CO}}_{2}\mathrm{Hb}$ absorption spectrum was measured for the first time. Such spectrum being close to that of HHb, the use of a theoretical background based on pulse oximetry theory for noninvasive ${\mathrm{Pa}}_{{\mathrm{CO}}_{2}}$ measurement seems extremely challenging.

## 1 Introduction

The accurate determination of blood gases—namely dioxygen ($O_{2}$) and carbon dioxide (${\mathrm{CO}}_{2}$)—is of crucial importance in medical care since it gives circulatory as well as ventilatory clues on the state of a patient.^1^ In particular, in case of acute respiratory failure, chronic obstructive pulmonary disease or cystic fibrosis, the monitoring of parameters such as the arterial blood dioxygen saturation (${\mathrm{SatO}}_{2}$) and ${\mathrm{CO}}_{2}$ arterial pressure (${\mathrm{Pa}}_{{\mathrm{CO}}_{2}}$) can strongly affect patient handling and outcome.^2^^–^^4^

Pulse oximetry makes it possible to determine ${\mathrm{SatO}}_{2}$. It is cheap, noninvasive, accurate, and as such, is widely used in clinical context.^5^^,^^6^ At the opposite, ${\mathrm{Pa}}_{{\mathrm{CO}}_{2}}$ measurements methods are far less satisfactory. In a nutshell, they consist of arterial blood sampling, airway capnometry, and transcutaneous capnometry.

Arterial blood sampling is the gold standard for ${\mathrm{Pa}}_{{\mathrm{CO}}_{2}}$ measurement.^7^ However, it is invasive, can be both painful and risky,^8^ needs expensive blood gas analyzer, and the blood samples must be promptly analyzed upon collection.^9^ To circumvent these flaws, two capnometry methods were developed. Airway capnometry—also known as capnography—is also invasive since it requires the patient to wear a mask or to undergo endotracheal intubation, and it is not reliable in case of end-tidal partial ${\mathrm{CO}}_{2}$ pressure $({\mathrm{Pet}}_{{\mathrm{CO}}_{2}})/{\mathrm{Pa}}_{{\mathrm{CO}}_{2}}$ mismatch caused by an increase in physiologic dead space.^10^^,^^11^ Finally, transcutaneous capnometry is derived from the Stow–Severinghaus electrode^12^ and has been used for a long time in the clinical practice.^13^ Its drawbacks are mainly the need to heat the skin of the patient between 37°C and 44°C, the need for frequent recalibrations of the sensor, and the high price of transcutaneous ${\mathrm{CO}}_{2}$ monitors (∼15k€). Consequently, there is a strong need for an alternative to the existing approaches, so as to provide a cheap, noninvasive, and accurate technique for long-term ${\mathrm{Pa}}_{{\mathrm{CO}}_{2}}$ monitoring.

The determination of ${\mathrm{SatO}}_{2}$ with pulse oximetry is made possible by the spectral differences existing between oxyhemoglobin ($O_{2}\mathrm{Hb}$) and deoxyhemoglobin (HHb), allowing to quantify their proportion in arterial blood. It would be extremely interesting if a similar technique was available to distinguish between carbamino-hemoglobin (${\mathrm{CO}}_{2}\mathrm{Hb}$) and HHb based on a difference of absorption between these two compounds. Since an equilibrium exists among blood pH, ${\mathrm{CO}}_{2}\mathrm{Hb}$ content, bicarbonate concentration, and ${\mathrm{Pa}}_{{\mathrm{CO}}_{2}}$,^14^^,^^15^ the determination of ${\mathrm{CO}}_{2}\mathrm{Hb}$ blood content would be a first step toward ${\mathrm{Pa}}_{{\mathrm{CO}}_{2}}$ determination.

Under such perspective, the starting point is to analyze the ${\mathrm{CO}}_{2}\mathrm{Hb}$ absorption spectrum. However—at least to the best of our knowledge—its measurement has never been reported in the literature. Consequently, the present article exposes the measurement and analysis of the ${\mathrm{CO}}_{2}\mathrm{Hb}$ absorption spectrum. The feasibility of pulse carbametry, the equivalent of pulse oximetry, substituting ${\mathrm{CO}}_{2}\mathrm{Hb}$ to $O_{2}\mathrm{Hb}$ are also assessed.

## 2 Materials and Methods

### 2.1 Hemoglobin Preparation and Measurement

Although ${\mathrm{CO}}_{2}\mathrm{Hb}$ absorption spectrum has yet to be measured, a number of authors were interested in measuring the spectra of other hemoglobin species in the past decades. The most complete work on the topic is undoubtedly that of Zijlstra et al.,^16^ which summarized almost a century of research aiming at measuring mainly $O_{2}\mathrm{Hb}$, HHb, met-hemoglobin (MetHb), and carboxy-hemoglobin (COHb) spectra. Inspired by their work, the following experimental protocol was elaborated:

– Human blood was diluted at 1:10 or 1:1000 [HEPES 20 mM, KCL 150 mM, pH 7.20, ethylene diamine tetraacetic acid (EDTA) 0.61 mM for the 1:10 dilution only, to prevent coagulation].
– Blood cells were lysed with ultrasound (Sonicator W-10, Heat Systems Ultrasonics).
– The obtained haemolysates were equilibrated using Eschweiler spherical glass tonometers during at least 30 min with either pure dinitrogen ($N_{2}$)—to obtain HHb—or pure ${\mathrm{CO}}_{2}$—to obtain ${\mathrm{CO}}_{2}\mathrm{Hb}$. Alternatively, they were let in ambient air so as to obtain $O_{2}\mathrm{Hb}$.
– The equilibrated hemolysates were carefully handled so as not to spoil them with ambient air. Syringes and cuvettes were rinsed three times with the tonometry gas, and the solutions were collected from the tonometers through a septum.
– Equilibrated solutions were poured in airtight quartz cuvettes (CV10Q1400FS, Thorlabs, 10 mm transmitted path length) placed inside the spectrophotometer (Carry 5000, Agilent Technologies).
– Measurements were performed on the 235- to 600-nm range [hereafter referred to as the ultraviolet/visible (UV–Vis) range] for the 1:1000 dilution and on the 600- to 1000-nm range [visible/infrared (Vis–IR) range] for the 1:10 dilution for $N_{2}$- and ${\mathrm{CO}}_{2}$-equilibrated solutions (235 to 590 nm and 590 to 1000 nm for air-equilibrated solutions).

**Fig. 1:**
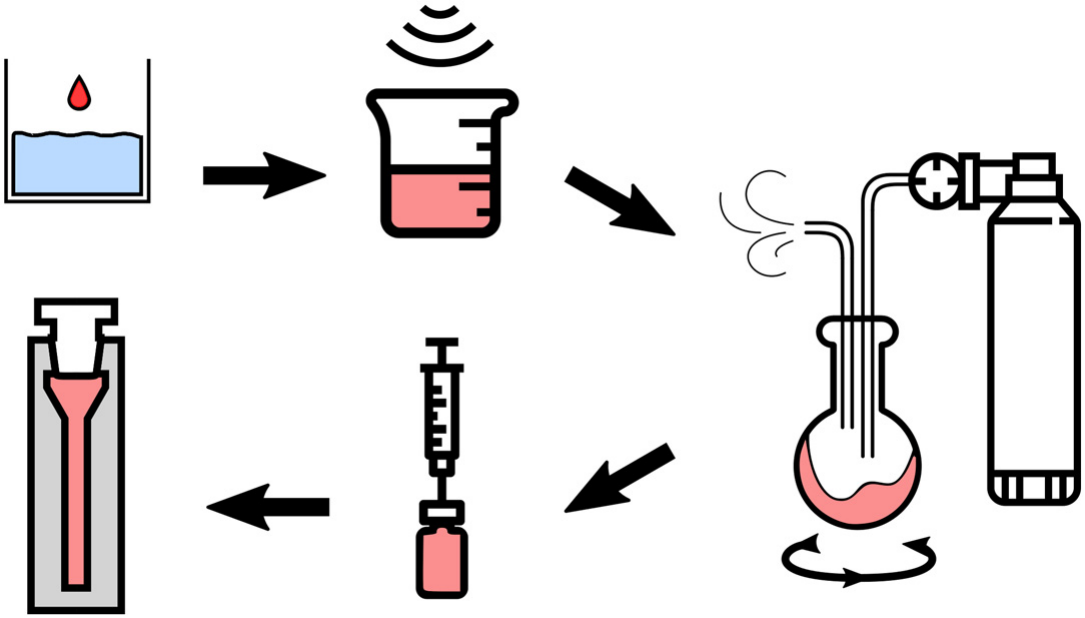
Preparation of blood, from the vein to the spectrophotometer. The different steps are (from top left to bottom left): venous sampling, dilution, ultrasound lysis, tonometry with ${\mathrm{CO}}_{2}$ or $N_{2}$, careful handling, pouring into airtight glass cuvettes for spectrophotometric measurements.

Figure 1 shows the above-described steps, which led to six different mixtures: diluted lysed blood at 1:10 or 1:1000 ratio, equilibrated with ${\mathrm{CO}}_{2}$, $N_{2}$, or ambient air. In addition, the dilution medium was also measured: pure for baseline correction, and with EDTA and sodium metabisulfite separately, to assess the influence of these substances on the hemolysates measurements. Indeed, no information about the absorption spectrum of sodium metabisulfite nor the one of EDTA in solution is to be found in the literature. An analysis of the relative variations of the spectrum of the dilution medium with and without sodium metabisulfite and EDTA was thus performed. Concentrations in the intramedium were 6.1 mM for EDTA and 2.0 mM for sodium metabisulfite.

In addition, the influence of tonometry duration on the obtained spectra was investigated. Equilibration durations were varied between 30 and 45 min, in 3 min steps, whereas measuring the absorbance at the 759-nm peak of the spectra of ${\mathrm{CO}}_{2}\mathrm{Hb}$ and HHb. A Pearson correlation coefficient test was then performed, searching for an influence of the tonometry duration on the measured absorbance values.

### 2.2 Assessment of Pulse Carbametry Feasibility

For pulse carbametry to be achievable, one needs that the absorption spectra of ${\mathrm{CO}}_{2}\mathrm{Hb}$ and HHb exhibit sufficient differences at accessible wavelengths. Assessing how much is sufficient is a complex task, which can be answered by addressing the following questions in order:

1. Are the measured average spectra computed for HHb and ${\mathrm{CO}}_{2}\mathrm{Hb}$ different?
2. If they are, is this difference statistically significant?
3. Does this difference—observed in the laboratory—translate into something actually exploitable in real life setups, where absorption measurements would be affected by:
  – additional absorption caused by tissues surrounding the blood vessels,
  – light reflection and scattering in the said tissues,
  – intersubject variations in physiology,
  – noises related to ambulatory measurement setups (ambient light and motion),
  – the accuracy of embedded sensors, which is bound to be lower than that of laboratory spectrophotometers.

Different methods were developed to answer each of these questions, as detailed below.

#### 2.2.1 Comparison of average spectra for Hb and CO_2_Hb

After offset correction and outlier removal, a set of spectra for each hemoglobin species was obtained. Each of these sets was then averaged to obtain the final absorption spectrum of the corresponding hemoglobin species. Resulting average spectra were then plotted for visual comparison (see Fig. 3).

#### 2.2.2 Statistical significance of the difference between spectra

The problem of assessing whether two absorption spectra are statistically different is extremely complex. Indeed, one has to deal with limitations of two kinds:

1. The estimated absorption spectrum of each species is a vector of dependent random variables A^species(λ) such that ∀  λ, A^species(λ)∼N[Aspecies(λ),σspecies(λ)2] where $\sigma_{\text{species}}(\lambda )$ is the standard variation of the noise on the measurement of the absorption of the species under consideration at wavelength λ. Thus, deciding whether the difference in the measured spectra of HHb and ${\mathrm{CO}}_{2}\mathrm{Hb}$ is statistically significant yields a multivariate analysis problem. Solving the latter would need one to perform at least as many measurements as there are points in the measured spectra (i.e., several hundreds).^17^
2. Variations in the measured spectrum of each species are bound to be induced by the slight differences in the manipulations specific to each species; thus, a statistically significant difference between measured spectra could actually reveal a difference in the protocol and not the spectra themselves.

To address such difficulties, the following analysis was performed on the measured hemoglobin spectra: for each pair of hemoglobin species (HHb, ${\mathrm{CO}}_{2}\mathrm{Hb}$) and (HHb, $O_{2}\mathrm{Hb}$), we computed the average, minimum, and maximum relative difference of their absorption spectra. This difference, computed for each wavelength, allows one to assess whether there is an exploitable discrepancy between the spectra of HHb and ${\mathrm{CO}}_{2}\mathrm{Hb}$ in the same order of magnitude as that between HHb and $O_{2}\mathrm{Hb}$.

#### 2.2.3 Applicability in real life setups

To estimate how much difference should be measurable between the spectra of HHb and ${\mathrm{CO}}_{2}\mathrm{Hb}$ for it to be exploitable in a pulse carbametry context, parallels were drawn with pulse oximetry. Specifically, we propose to

– use the literature available on pulse oximetry to obtain an estimation of the accuracy of photoplethysmographic measurements on human skin,
– derive a theoretical background for pulse carbametry, and
– use the aforementioned accuracy in such background along with the results of our measurement, to conclude on the feasibility of pulse carbametry.

Note that we focus, in all our analysis, on a two-wavelength system. Even though we acknowledge that multiwavelength approaches can improve the performance of photoplethysmographic systems, the goal of this paper is to evaluate whether there are couples of wavelengths for which the spectra of HHb and ${\mathrm{CO}}_{2}\mathrm{Hb}$ exhibit exploitable difference. The optimization of the exploitation of these pairs of wavelengths, should they exist, is out of the scope of this paper.

##### Accuracy in pulse oximetry

Our goal here is not to dive deeply into the theoretical foundations of pulse oximetry—as it has been done elsewhere^18^^–^^21^—but to estimate the sources of inaccuracy of this technique. In pulse oximetry, ${\mathrm{SatO}}_{2}$ is derived from the so-called ratio of ratio, R, which in turns utilizes absorbance of light by human tissues at two different wavelengths. R is given as

$$R=\frac{E_{\mathrm{AC}}(\lambda_{1})/E_{\mathrm{DC}}(\lambda_{1})}{E_{\mathrm{AC}}(\lambda_{2})/E_{\mathrm{DC}}(\lambda_{2})}=\frac{E_{O_{2}\mathrm{Hb}}(\lambda_{1})\cdot {\mathrm{SatO}}_{2}+E_{\mathrm{HHb}}(\lambda_{1})\cdot (1-{\mathrm{SatO}}_{2})}{E_{O_{2}\mathrm{Hb}}(\lambda_{2})\cdot {\mathrm{SatO}}_{2}+E_{\mathrm{HHb}}(\lambda_{2})\cdot (1-{\mathrm{SatO}}_{2})},$$ (1)

where $\lambda_{1}$ and $\lambda_{2}$ are the two measurement wavelengths, often 660 and 940 nm, and E is either the extinction coefficient of the tissues, measured with photoplethysmography (PPG) and decomposed in its alternative (AC) and continuous (DC) parts (left hand side) or the extinction coefficient of hemoglobin species (right-hand side).

**Fig. 2:**
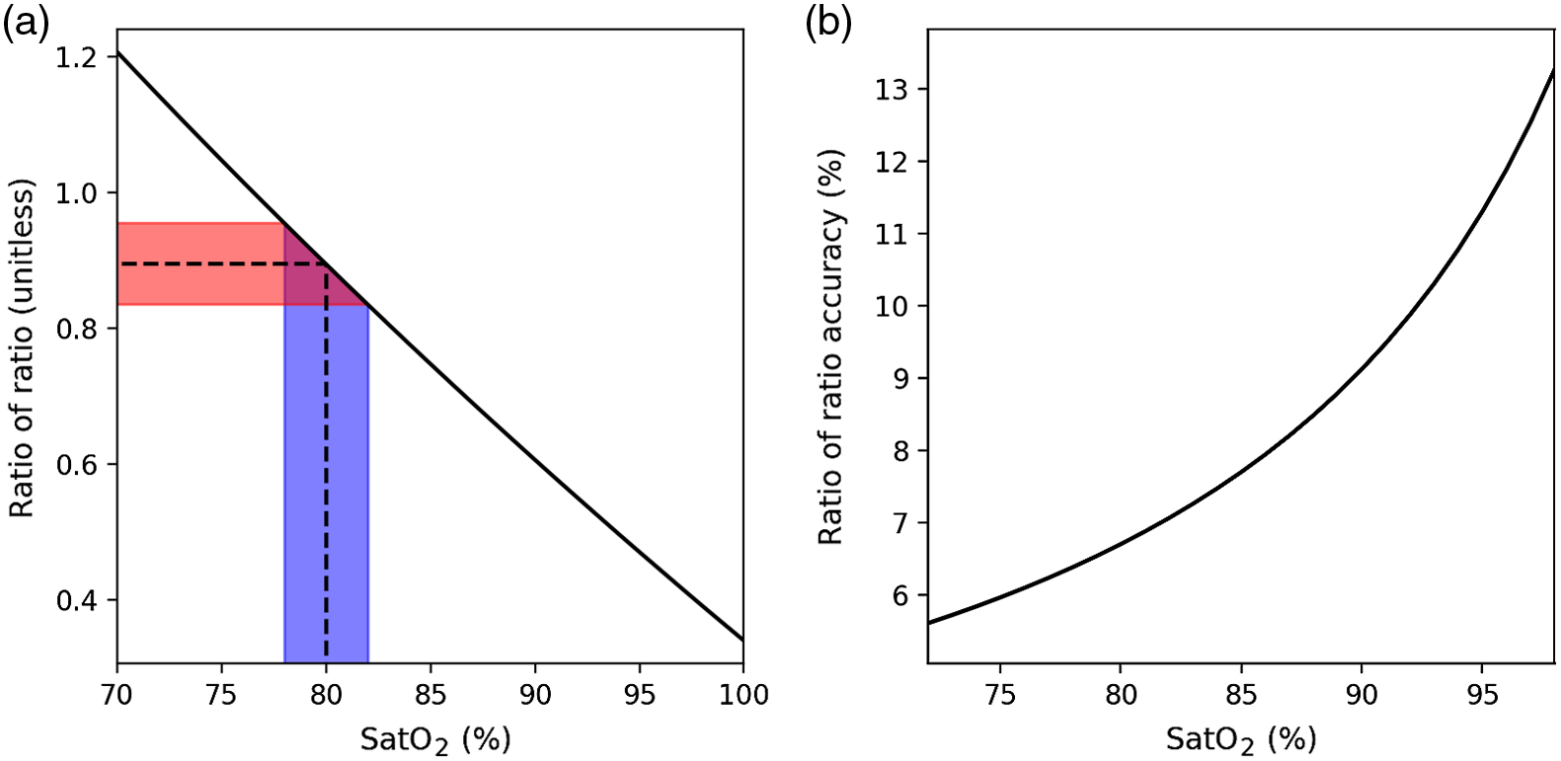
(a) An error of 2% on the ${\mathrm{SatO}}_{2}$ reading at 80% of ${\mathrm{SatO}}_{2}$ (in blue) is the result of an inaccuracy of 7% on the measurement of R (in red). (b) Conversely, if we impose a 2% error on the ${\mathrm{SatO}}_{2}$ reading, we can compute the relative variation of R, which generated such error.

The question at stake for our analysis is then: what accuracy on R is achievable in practice by common photoplethysmographic sensors? As can be seen from Eq. (1), such an accuracy on R can be estimated based on the accuracy on ${\mathrm{SatO}}_{2}$ that is reported in the literature. The latter has been studied in many clinical trials—themselves reviewed^5^^,^^6^—and a mean standard deviation of 2% on the 70%- to 100%-${\mathrm{SatO}}_{2}$ range can be considered to be achievable under good measurement conditions—that is, mainly no movement from the subject and a good perfusion index. Figure 2 shows the results obtained when taking such a standard deviation for ${\mathrm{SatO}}_{2}$ and computing the corresponding R measurement inaccuracies at the wavelengths 660 and 940 nm—the two most-used wavelengths in pulse oximetry—using Zijlstra et al.^16^ absorption coefficient for $O_{2}\mathrm{Hb}$ and HHb.

One can see that the lower the oxygen saturation is, the more accurate the measurement of R needs to be to guarantee a given accuracy on ${\mathrm{SatO}}_{2}$. The minimum measured accuracy on R is of 5.6% at 70% of ${\mathrm{SatO}}_{2}$. Such calculations were made with the hypothesis that all errors were Gaussians.

##### Accuracy in pulse carbametry

Such a minimum error in the case of pulse oximetry gives a best-case—hence optimistic—value of the achievable accuracy on R measurement, using transcutaneous ratiometric techniques. When applying a similar method to obtain the concentration in ${\mathrm{CO}}_{2}\mathrm{Hb}$ in a pulse carbametry context, one may thus expect—at best—a similar accuracy on R. Such a context will be detailed below.

Let us consider a binary system composed solely of HHb and ${\mathrm{CO}}_{2}\mathrm{Hb}$ and define carbon dioxide saturation (${\mathrm{SatCO}}_{2}$) as

$${\mathrm{SatCO}}_{2}=\frac{C_{{\mathrm{CO}}_{2}\mathrm{Hb}}}{C_{{\mathrm{CO}}_{2}\mathrm{Hb}}+C_{\mathrm{HHb}}},$$ (2)

where C is the concentration of the different species involved. Using the pulse oximetry theoretical background,^5^ ${\mathrm{SatCO}}_{2}$ can be derived from a measured ratio of ratio R as

$${\mathrm{SatCO}}_{2}(\lambda_{1},\lambda_{2})=\frac{E_{\mathrm{HHb}}(\lambda_{1})-R\cdot E_{\mathrm{HHb}}(\lambda_{2})}{E_{\mathrm{HHb}}(\lambda_{1})-E_{{\mathrm{CO}}_{2}\mathrm{Hb}}(\lambda_{1})-R\cdot [E_{\mathrm{HHb}}(\lambda_{2})-E_{{\mathrm{CO}}_{2}\mathrm{Hb}}(\lambda_{2})]}.$$ (3)

The feasibility of pulse carbametry will be assessed as follows: considering the extinction coefficients $E_{{\mathrm{CO}}_{2}\mathrm{Hb}}$ and $E_{\mathrm{HHb}}$ derived from our spectrophotometric measurements, and the aforementioned standard deviation on R, the standard deviation on ${\mathrm{SatCO}}_{2}$ will be computed for each ($\lambda_{1}$, $\lambda_{2}$) couple on the 235- to 1000-nm range. Then, finding the minimum value of this deviation with respect to ($\lambda_{1}$, $\lambda_{2}$) will provide the best achievable accuracy using pulse carbametry. More explicitly, the minimum ${\mathrm{SatCO}}_{2}$ accuracy reachable with pulse carbametry is given as

$$\delta {\mathrm{SatCO}}_{2}=\underset{\left(\lambda_{1},\lambda_{2}\right)}{\min}\;\sigma {\mathrm{SatCO}}_{2}(\lambda_{1},\lambda_{2}),$$ (4)

wherein $\sigma {\mathrm{SatCO}}_{2}(\lambda_{1},\lambda_{2})$ is the relative standard deviation of ${\mathrm{SatCO}}_{2}(\lambda_{1},\lambda_{2})$, computed from the R standard deviation in the case of pulse oximetry (5.6%). Judging whether a given minimal accuracy $\delta {\mathrm{SatCO}}_{2}$ is small enough is essentially an arbitrary choice. Still, one can rely on the clinically accepted range for ${\mathrm{Pa}}_{{\mathrm{CO}}_{2}}$, which is ±7.5  mmHg (95% limits of agreement, corresponding to ±2 S.D.).^22^ Such a range translates into a standard deviation of 9% on ${\mathrm{Pa}}_{{\mathrm{CO}}_{2}}$ reading, at a standard ${\mathrm{Pa}}_{{\mathrm{CO}}_{2}}$ level of 40 mmHg. Thus, the decision threshold on $\delta {\mathrm{SatCO}}_{2}$ was set to this value as a first approximation, i.e., if $\delta {\mathrm{SatCO}}_{2}$ is below 9%, pulse carbametry will be considered feasible.

## 3 Results

### 3.1 Hemoglobin Spectra

**Fig. 3:**
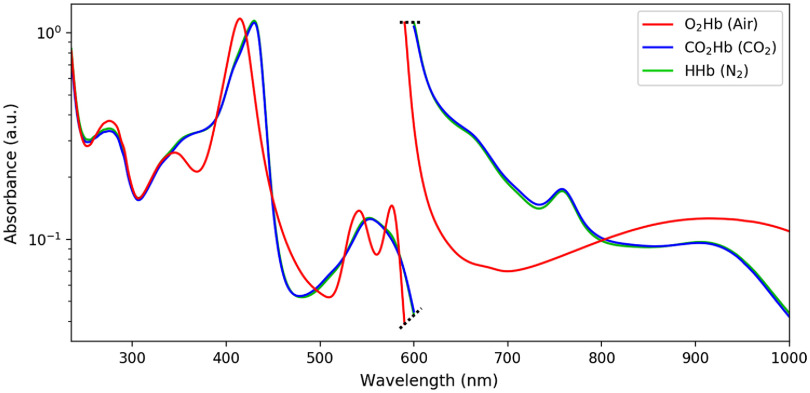
Measured absorption spectra of diluted lysed blood tonometered with $O_{2}$, $N_{2}$, or ${\mathrm{CO}}_{2}$. The vertical scale is arbitrary, data were scaled for representation and corresponds to two different dilution ratios of 1:1000 for the UV–Vis 235 to 600 nm range (left) and 1:10 for the Vis–IR 600- to 1000-nm range (right) (ranges are 235 to 590 nm and 590 to 1000 nm for air-equilibrated solutions). The black dashed line separates the UV–Vis from the Vis–IR measurements.

The obtained spectra of diluted lysed blood equilibrated with ambient air, pure $N_{2}$, and pure ${\mathrm{CO}}_{2}$ are presented in Fig. 3 after baseline correction and outlier removal. Each spectrum is averaged over (UV–Vis/Vis–IR spectra number): 24/13 (${\mathrm{CO}}_{2}$), 10/17 ($N_{2}$), and 32/38 (air) measurements. The variations in the number of trials are explained by several reasons. At first, there were two different measurement campaigns for the UV–Vis and Vis–IR range, leading to less measurements in the Vis–IR (some fluorescence measurements were performed instead, unpublished). Then, there were twice as much measurements made with $O_{2}\mathrm{Hb}$ as with $N_{2}$ or ${\mathrm{CO}}_{2}$, only because $O_{2}\mathrm{Hb}$ was readily available and measured while waiting for HHb and ${\mathrm{CO}}_{2}\mathrm{Hb}$ to be obtained by tonometry. Finally, we used an outlier removal algorithm based on the standard deviation of the measurements, removing measurements diverging more than roughly ±2.5 standard deviation from the mean, using an adaptative threshold and a recursive algorithm inspired by the work of Hadi et al.^23^. This threshold choice was arbitrary, as is that of outlier detection and removal in the general case.^24^ Given the important number of wavelengths in each measurement and the limited number of measurements performed, a power calculation was not feasible.

The absorption spectra of HHb and ${\mathrm{CO}}_{2}\mathrm{Hb}$ appear to be extremely close, especially compared to $O_{2}\mathrm{Hb}$. The standard deviations in absorption for $O_{2}\mathrm{Hb}$, ${\mathrm{CO}}_{2}\mathrm{Hb}$, and HHb were (mean standard deviation, minimum, and maximum) $1.20_{0.38}^{7.55}\;\mathrm{mAbs}$, $3.16_{0.51}^{36.34}\;\mathrm{mAbs}$, and $2.43_{0.40}^{21.02}\;\mathrm{mAbs}$, corresponding to relative variations of $0.53_{0.14}^{2.24}\%$, $1.08_{0.05}^{5.74}\%$, and $0.81_{0.07}^{4.59}\%$, respectively.

**Fig. 4:**
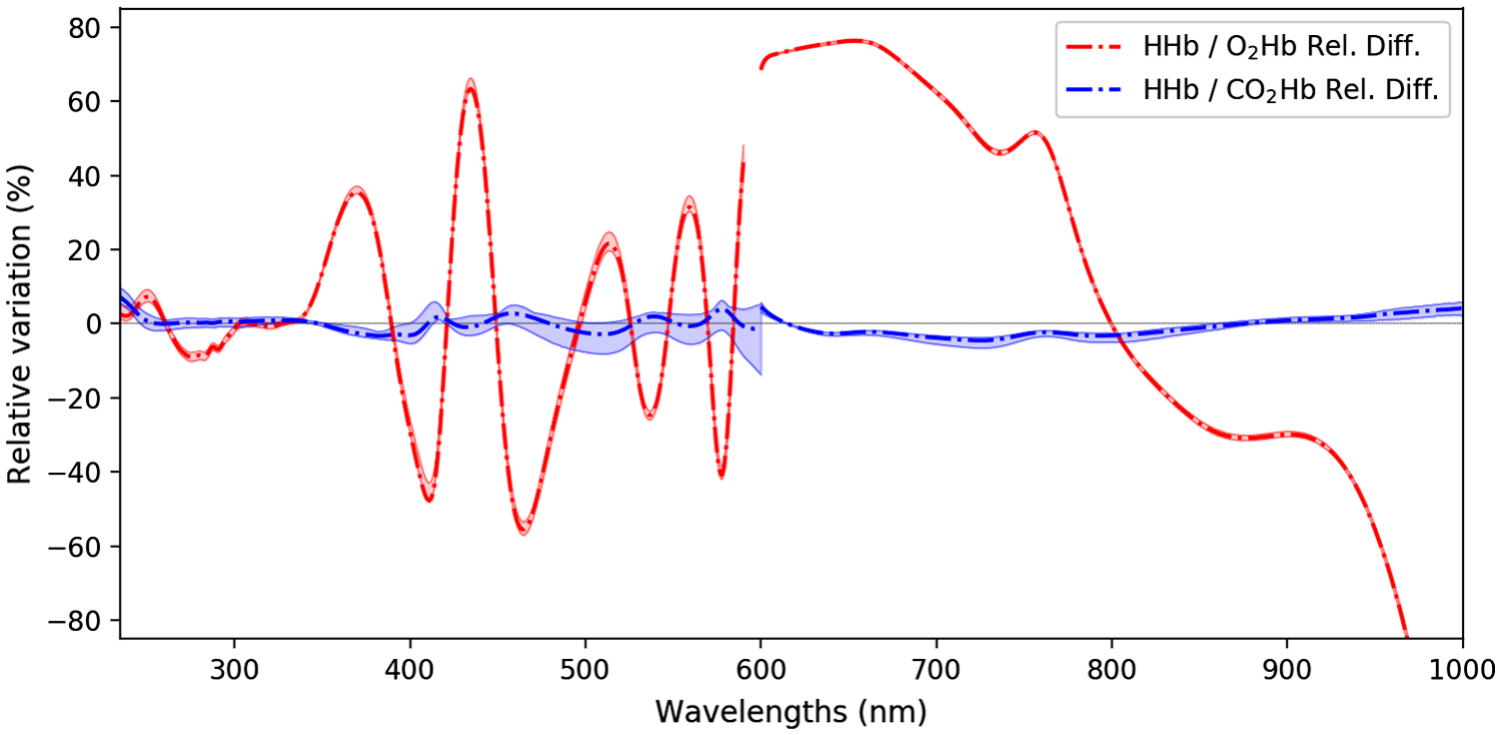
Relative differences between HHb and ${\mathrm{CO}}_{2}\mathrm{Hb}$ (in blue) and between HHb and $O_{2}\mathrm{Hb}$ (in red) absorption spectra. The two thin lines for each comparison represent the maximum and minimum values along all measurements. For instance, for $\mathrm{HHb}/{\mathrm{CO}}_{2}\mathrm{Hb}$ comparison, at a given wavelength, the higher line represents HHb maximum value minus ${\mathrm{CO}}_{2}\mathrm{Hb}$ minimum value, and the lower line represents HHb minimum value minus ${\mathrm{CO}}_{2}\mathrm{Hb}$ maximum value.

Figure 4 gives a more quantitative analysis to the difference among $O_{2}\mathrm{Hb}$, ${\mathrm{CO}}_{2}\mathrm{Hb}$, and HHb spectra. While the relative differences between $O_{2}\mathrm{Hb}$ and HHb spectra—clearly visible in Fig. 3—reach several tens of percent, those between ${\mathrm{CO}}_{2}\mathrm{Hb}$ and HHb are much more tenuous. The differences between mean $O_{2}\mathrm{Hb}$ and HHb spectra on the one hand and ${\mathrm{CO}}_{2}\mathrm{Hb}$ and HHb spectra on the other hand were (mean absolute difference and maximum) $0.18^{\left(1.99\right)}\;\mathrm{Abs}$ and $0.08^{\left(0.11\right)}\;\mathrm{Abs}$, corresponding to relative variations of $36^{\left(153\right)}\%$ and $2^{\left(13\right)}\%$, respectively.

### 3.2 Intra Medium

**Fig. 5:**
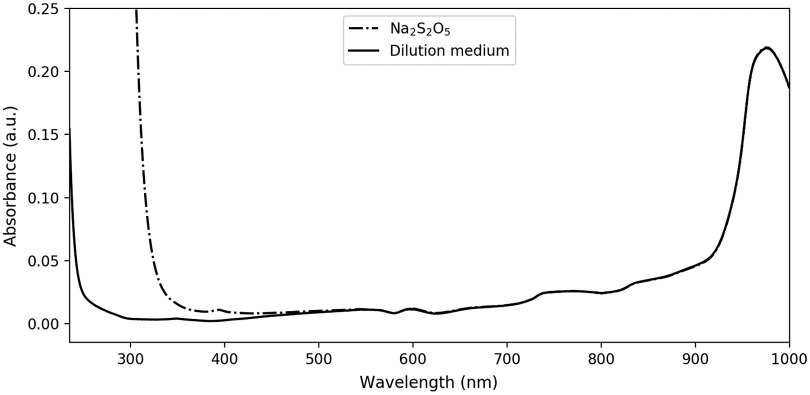
Absorption spectra of the dilution medium with and without the addition of sodium metabisulfite (2.0 mM), we can observe a marked absorbing effect of sodium metabisulfite in the ultraviolet, up to almost 500 nm.

**Fig. 6:**
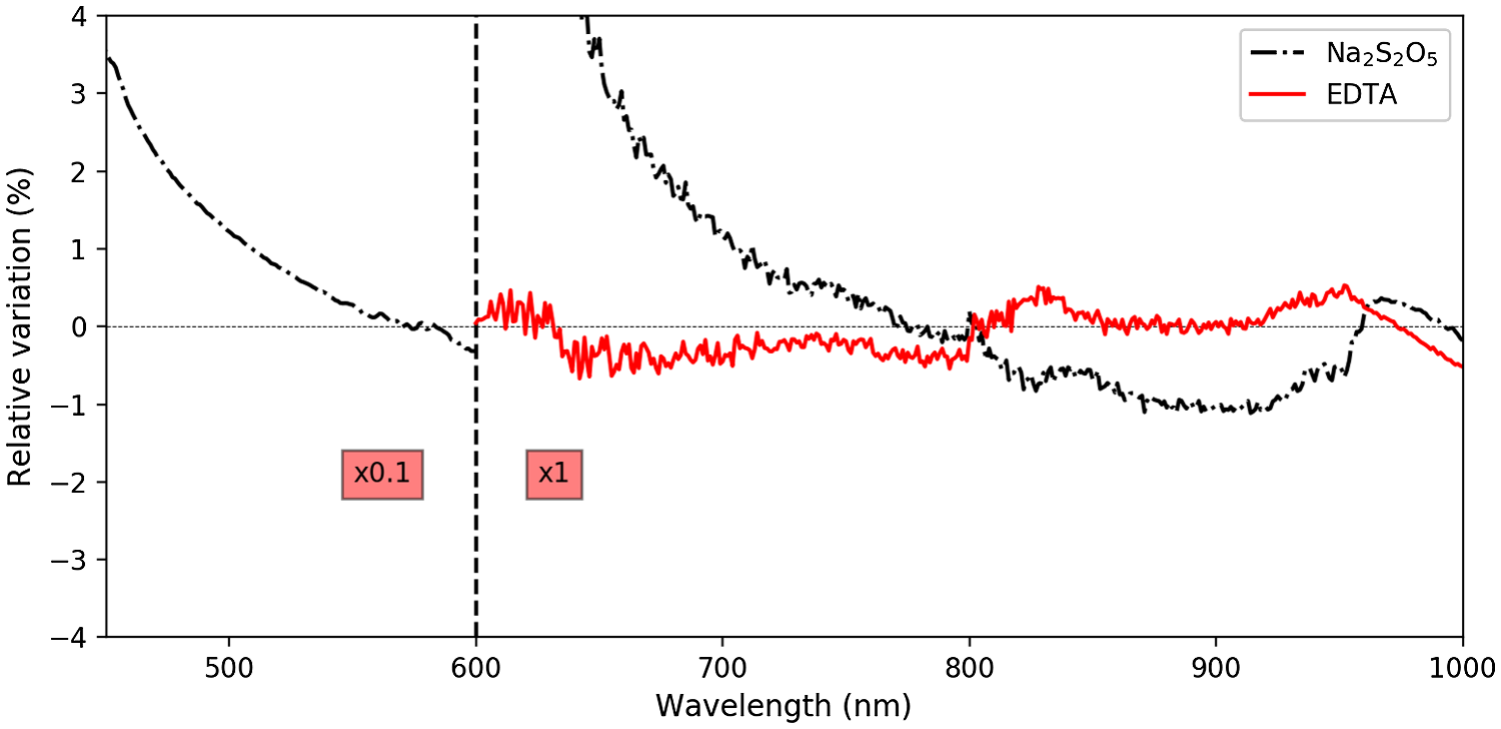
Relative variations of the dilution medium absorption spectrum upon the addition of EDTA (0.61 mM) or sodium metabisulfite (2.0 mM). The left side is multiplied by 0.1 (∼10% of relative variation for sodium metabisulfite at 500 nm).

Figure 5 shows the spectra of the dilution medium with and without sodium metabisulfite. Results with EDTA are not shown since they were indistinguishable from pure dilution medium on a full scale view. More subtle effects of these substances are shown in Fig. 6, which focuses on the deviation—with respect to pure dilution medium—of the prepared EDTA or sodium metabisulfite solutions. Again, each spectrum is averaged over several measurements. For the UV–Vis/Vis–IR range: 28/34 (pure) and 12/11 (sodium metabisulfite) measurements. For the Vis–IR only range: five (EDTA) measurements.

### 3.3 Tonometry Duration

The influence of tonometry duration on the measured absorbances was found to be insignificant for durations between 30 and 45 min, with test results being ρ=−0.13, p=0.62, on 17 samples for $N_{2}$ tonometry and ρ=−0.04, p=0.91, on 13 samples for ${\mathrm{CO}}_{2}$ tonometry (Pearson correlation coefficient). We concluded that 30 min of equilibration time were enough to obtain either ${\mathrm{CO}}_{2}\mathrm{Hb}$ or HHb.

### 3.4 Pulse Carbametry

**Fig. 7:**
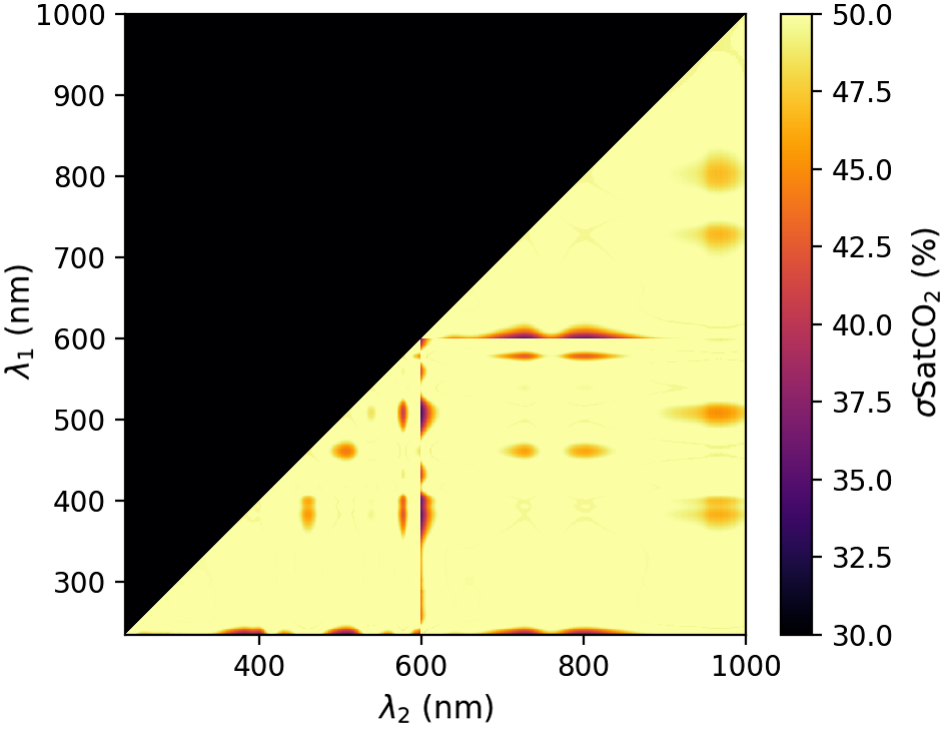
$\sigma {\mathrm{SatCO}}_{2}$ as a function of $\left(\lambda_{1},\lambda_{2}\right)$ on the 235- to 1000-nm range.

The obtained ${\mathrm{CO}}_{2}\mathrm{Hb}$ and HHb spectra—${\mathrm{CO}}_{2}$ and $N_{2}$ tonometry, respectively—shown in Fig. 3 were then used to perform the analysis presented in Sec. 2.2.3. The value of $\sigma {\mathrm{SatCO}}_{2}$ as a function of the two wavelengths $\left(\lambda_{1},\lambda_{2}\right)$ is shown in Fig. 7.

The minimal value taken by $\sigma {\mathrm{SatCO}}_{2}$ is $\delta {\mathrm{SatCO}}_{2}=34.2\%$ at $\lambda_{1}=508\;\mathrm{nm}$ and $\lambda_{2}=600\;\mathrm{nm}$. However, as can be seen from the asymmetry of the spots on the $\lambda_{1}=600\;\mathrm{nm}$ or $\lambda_{2}=600\;\mathrm{nm}$ lines, these values likely originate from limitation of the measurement system. Indeed, with a 1:1000 dilution, lysed blood absorption was below 1.2 Abs on the full 235- to 600-nm range. At the opposite, the 1:10 dilution topped at 2.8 Abs near 600 nm, which is close to the saturation value of 3.0 Abs of the spectrophotometer that we used. Ideally, to circumvent this flaw of the measurement setup, three dilutions at 1:10, 1:100, and 1:1000 could have been performed on the 235 to 450 nm, 450 to 650 nm, and 650 to 1000 nm ranges, respectively.

Still, even with these flaws, which—in the worst case—could increase the spectral differences between HHb and ${\mathrm{CO}}_{2}\mathrm{Hb}$ and thus lower the error on ${\mathrm{SatCO}}_{2}$ measurements, the reached accuracy is still far above the 9% target established previously. If $\sigma {\mathrm{SatCO}}_{2}$ values near the 600-nm lines are discarded and focus is made on other spots such as the ($\lambda_{1}=510\;\mathrm{nm}$, $\lambda_{2}=580\;\mathrm{nm}$) area, values above 41% are to be found for $\delta {\mathrm{SatCO}}_{2}$.

## 4 Discussion

At first, the chosen method for obtaining ${\mathrm{CO}}_{2}\mathrm{Hb}$, HHb, and $O_{2}\mathrm{Hb}$ is discussed. Then, the hemoglobin spectra are compared with those available in the literature, the influence of EDTA and sodium metabisulfite are also discussed. Finally, the feasibility of pulse carbametry is assessed.

### 4.1 On the Chosen Method

Among the several authors who measured hemoglobin absorption spectra, some used freshly drawn blood^16^^,^^25^^–^^33^ whereas others preferred lyophilized hemoglobin.^34^^–^^36^ Lyophilized blood, despite its convenience, has several drawbacks. First, it is composed merely of MetHb^33^^,^^37^ and thus needs an oxidation procedure to convert it back into $O_{2}\mathrm{Hb}$, a step that involves chemicals that might interfere with the hemoglobin spectrum. Second, a better affinity of hemoglobin has been reported for hemoglobin extracted from freshly drawn blood.^25^^,^^33^ These considerations drove our choice toward fresh blood as a hemoglobin source.

However, fresh blood requires the lysis of erythrocytes and other blood cells to yield a limpid solution. Despite the common use of a surfactant such as Sterox SE^27^^,^^28^^,^^32^ or equivalent,^25^^,^^29^^,^^31^ we preferred an ultrasound lysis, which adds no foreign chemical in blood for the same effect.^30^

Fresh blood sampling could also require centrifugation to keep only the erythrocytes and avoid spectral interferences from other blood components, namely leucocytes and lipids. Yet, since hemoglobin is the main absorbing compound in blood, by two up to three orders of magnitude,^38^ we did not consider the centrifugation step mandatory. Lastly, fresh blood sampling requires the addition of an anticoagulant if it is not largely diluted. Consequently, for the 1:10 dilution ratio—on the 600- to 1000-nm range—we considered the addition of EDTA to the collected blood. Its spectral influence was measured and found to be negligible on the studied range—as shown in Fig. 6—with relative absorption variations in the ±1% range, corresponding to absolute variations in the ±1  mAbs range, well below the measurement standard deviation ($1.24_{-9.3}^{+14.1}\;\mathrm{mAbs}$ for the dilution medium itself for instance, similar values were found for dilution medium with EDTA). We concluded that EDTA did not have any influence on the measured spectra, in the quantity used in our experiments.

Concerning hemoglobin measurements in its reduced form, the use of sodium dithionite (${\mathrm{Na}}_{2}S_{2}O_{4}$) has been reported, as a mean to quickly obtain HHb.^26^^,^^27^^,^^30^^,^^33^^,^^36^ Alas, it has also been reported to alter its absorption spectrum.^16^^,^^35^^,^^39^ We conducted investigations on the possible use of sodium metabisulfite (${\mathrm{Na}}_{2}S_{2}O_{5}$), which—like sodium dithionite—yields to the production of aqueous bisulfite anion, the strong reducing agent converting $O_{2}\mathrm{Hb}$ into HHb. Our results—see Figs. 5 and 6—confirm earlier observations and extend them with quantitative measurements on the 235- to 1000-nm range. We would not recommend the use of bisulfite anion for its strong absorption, especially in the short wavelengths up to 500 nm.

Consequently, tonometry was chosen to obtain HHb and ${\mathrm{CO}}_{2}\mathrm{Hb}$. Concerning its duration, 30 min was found to be sufficient in Eschweiler spherical glass tonometers filled with 6 mL of diluted lysed blood as demonstrated in Sec. 3.3. However, one should bear in mind that this duration is strongly dependent on several parameters, such as the shape of the tonometer used for equilibration, its filling level, or the gas flow rate for instance.

The dilution medium was chosen to correspond to an intracellular medium (with high $K^{+}$ concentration). pH was also set to an erythrocyte intracellular value of 7.2 since it has been reported to be that of the inner erythrocytes.^40^^,^^41^ It has also been reported^35^^,^^42^^,^^43^ that the pH has an impact—although relatively small—on the measured hemoglobin spectra. Concerning the choice of HEPES as a buffer, a better preservation of the hemoglobin oxygenation function was reported with HEPES over Tris/Bis-Tris.^44^ Finally, the chosen dilution ratios are justified since hemoglobin has been reported to follow Beer–Lambert law, would it be for extremely diluted or concentrated solutions.^34^

### 4.2 Hemoglobin Spectra

**Fig. 8:**
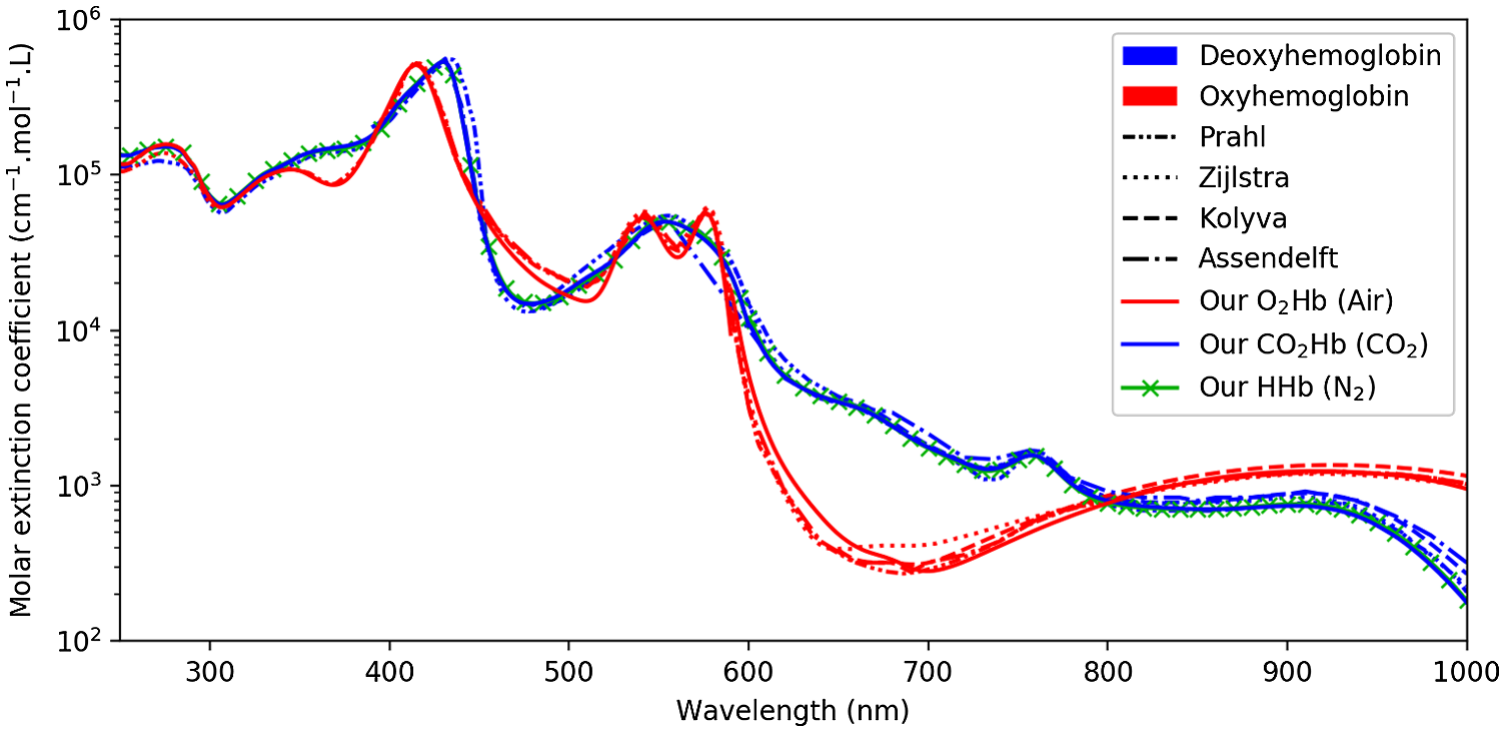
Our measurements (plain line) compared to that of Prahl,^45^ Zijlstra et al.,^16^ Kolyva et al.,^46^ and Assendelft.^27^ Our measurements are consistent with that of the literature for $O_{2}\mathrm{Hb}$ (air) and HHb ($N_{2}$).

The measured spectra of diluted lysed blood, either equilibrated with $N_{2}$ or ambient air, are extremely close to that of the literature, as can be seen in Fig. 8. This comforts us in the method that we employed and the above-mentioned choices. The small discrepancies observed between our spectra and that of the literature may be explained by a number of methodological differences. For instance, several authors^27^^,^^28^^,^^32^ used a surfactant such as Sterox SE to perform the erythrocyte lysis, whereas we chose to use ultrasound. The surfactant may have a spectral impact, which—to the best of our knowledge—has never been quantified. Other authors, even when choosing tonometry, added some sodium dithionite before measuring.^16^ Yet, sodium dithionite, such as sodium metabisulfite, is known to have a marked spectral influence.^35^ Zijlstra et al.^16^ also mentioned that in case of too long tonometry, supernatant residues sometimes appeared in the diluted blood, the nature of which was not determined. Although we did not observe such behavior, a small turbidity might have been present in their measurements, or ours. Of all the literature spectra shown in Fig. 8, Prahl and Kolyva only offered raw coefficients, whereas Assendelft and Zijlstra detailed their protocol. It is therefore difficult to analyze the potential sources of differences between their spectra or between their spectra and ours.

Overall, the repeatability of the measurements was fairly good, with mean standard deviation of 0.53%, 1.08%, and 0.81%, for $O_{2}\mathrm{Hb}$, ${\mathrm{CO}}_{2}\mathrm{Hb}$, and HHb measurements, respectively, which can be compared to values between 0.8% and 2.1% reported by Zijlstra et al.^16^ The rather high maximum standard deviations reported in Sec. 3.1 (2.24%, 5.74%, and 4.59% for $O_{2}\mathrm{Hb}$, ${\mathrm{CO}}_{2}\mathrm{Hb}$, and HHb, respectively) are mainly due to the measurement limits of the spectrophotometer near 600 nm, corresponding to either too low (≤0.05  Abs) absorbance below 600 nm or too high (≥2.5  Abs) absorbance above 600 nm. When computing the mean standard deviation without the 580- to 620-nm range for $O_{2}\mathrm{Hb}$, ${\mathrm{CO}}_{2}\mathrm{Hb}$, and HHb, maximum standard deviations values drop to 7.25, 12.60, and 21.02 mAbs, corresponding to relative variations of 1.31%, 2.78%, and 2.54%, respectively. The mean absorption spectra that we measured for O_2_Hb, CO_2_Hb, and HHb are published as Supplementary Material (see also the supplemental “readme” file explaining the units used).

Fig. 3 also reveals that the spectra of HHb and ${\mathrm{CO}}_{2}\mathrm{Hb}$ are extremely close, their differences being more than one order of magnitude below the one between the spectra of $O_{2}\mathrm{Hb}$ and HHb, as can be seen in Fig 4. Moreover, the measured HHb and ${\mathrm{CO}}_{2}\mathrm{Hb}$ spectra are similar to the HHb spectra already available in the literature, as can be seen in Fig. 8. Such observations tend to make one believe that the formation of carbamined compounds between ${\mathrm{CO}}_{2}$ and hemoglobin terminal amine groups does not change the hemoglobin molecule conformation significantly, hence bringing no spectral alteration. However, such intuition shall not have the value of evidence, this is why the possible use of slight differences between HHb and ${\mathrm{CO}}_{2}\mathrm{Hb}$ will now be discussed.

### 4.3 Pulse Carbametry

Given the accuracy threshold that was fixed at 9% for $\delta {\mathrm{SatCO}}_{2}$, and the observed value of 34.2%—or even more if the values close to the spectrophotometer saturation limit are discarded—we could readily conclude that pulse carbametry—as it was presented—is not feasible. However, several additional aspects of this technique need to be further discussed.

At first sight, the consideration of a binary system composed solely of HHb and ${\mathrm{CO}}_{2}\mathrm{Hb}$ can seem surprising. Indeed, in practice, human arterial blood is composed at least of $O_{2}\mathrm{Hb}$, HHb, and ${\mathrm{CO}}_{2}\mathrm{Hb}$—and even COHb^47^^,^^48^ and MetHb^49^^,^^50^ in small amounts. That being said, it should be clear that the demonstrated inability to distinguish between ${\mathrm{CO}}_{2}\mathrm{Hb}$ and HHb, even when they are the only absorbing compounds involved, would be worsened by the addition of any other perturbing absorbing species— e.g., $O_{2}\mathrm{Hb}$. An in-depth analysis of the tertiary system $O_{2}\mathrm{Hb}$–HHb–${\mathrm{CO}}_{2}\mathrm{Hb}$ would have been necessary only if pulse carbametry had been found to be possible in a binary system.

Then, we made the hypothesis that all studied errors were Gaussian. It is most often considered to be the case in the literature,^5^^,^^6^ and we will also stick to this hypothesis in the absence of evidence to the contrary. Thus, the main remaining question is to know whether the mathematical functions giving $R=f({\mathrm{SatO}}_{2})$ and ${\mathrm{SatCO}}_{2}=g(R)$ can be reasonably linearly approximated. The latter assumption has to be checked for f at the 660/940  nm couple, and for g on the full 235 to 1000 nm range. The calculation of the derivative of these two functions is straightforward and allows one to conclude that the relative variation of the slope of f stays below 3.8% on the 70- to 100%-${\mathrm{SatO}}_{2}$ range at 660/940  nm, whereas the slope of g stays below 2.2% on the 0% to 100%-${\mathrm{SatCO}}_{2}$ range at 340/600  nm (maximal value on the 235- to 1000-nm range). We can thus safely conclude that approximating f and g with linear functions is reasonable and that our hypothesis concerning Gaussian errors is justified.

Next, all the calculations leading to Fig. 7 were made considering a monochromatic skin illumination. In a typical pulse carbametry application, the light source is more likely to be a laser, laser diode, or light-emitting diode (LED). In such cases, the effect of a nonmonochromatic light source will be a degradation of $\delta {\mathrm{SatCO}}_{2}$ caused by the spectral spread of the source. Such spread will basically smooth HHb and ${\mathrm{CO}}_{2}\mathrm{Hb}$ spectra by convolving them with the emission spectrum of the source. Such spectrum can in turn be roughly regarded as a Gaussian window of full-width at half-maximum (FWHM) of a few (laser sources) or a few tenths (LED) of nanometers. For instance, a source with an FWHM of 5 nm leads to a $\delta {\mathrm{SatCO}}_{2}$ of 36.8%, with an FWHM of 20 nm this value reaches 42.1%, to be compared with the 34.2% of the aforementioned ideal monochromatic case.

Finally, the last assumption that we made concerns the extrapolation to pulse carbametry of the accuracy on R measurement in the ${\mathrm{SatO}}_{2}$ case. Such an assumption was made considering that the 660- and 940-nm wavelengths were not chosen randomly but to maximize pulse oximetry sensitivity. In other words, they were chosen such that a small change in ${\mathrm{SatO}}_{2}$ translates into a huge change in measured light intensity at certain wavelengths,^27^^,^^51^ but also such that they were in the tissues optical window—the 700- to 1000-nm range.^52^^–^^54^ Such considerations make the ${\mathrm{SatO}}_{2}$ 660/940  nm situation a best case, and we would expect other wavelengths couples to give equally or worse accurate R measurements. This remains, however, a supposition since—to the best of our knowledge—there appears to be no study on the measurement accuracy on ${\mathrm{SatO}}_{2}$—and thus R—at wavelengths different from the usual 660/940  nm pair. Still, it is worth noticing that our conclusions would remain unchanged, even if we managed somehow to drastically reduce the measuring accuracy on R—say by a factor two or three, we would still have a $\delta {\mathrm{SatCO}}_{2}$ value above 9%.

## 5 Conclusion

In this study, ${\mathrm{CO}}_{2}\mathrm{Hb}$ absorption spectrum was measured for the first time. $O_{2}\mathrm{Hb}$, HHb, and ${\mathrm{CO}}_{2}\mathrm{Hb}$ were obtained from diluted lysed blood equilibrated with ambient air, pure $N_{2}$, and pure ${\mathrm{CO}}_{2}$, respectively. Their isolation method was discussed thoroughly, including the possible use of EDTA or sodium metabisulfite.

The absorption spectra of $O_{2}\mathrm{Hb}$ and HHb were close to that of literature, whereas the absorption spectrum of ${\mathrm{CO}}_{2}\mathrm{Hb}$ was extremely close to that of HHb. No influence of EDTA was found. Sodium metabisulfite, however, strongly absorbs in the ultraviolet and visible range up to 500 nm. As such, it should not be used in this spectral range for hemoglobin reduction.

A theoretical variation of pulse oximetry applied to the determination of ${\mathrm{CO}}_{2}\mathrm{Hb}$ fraction was presented, called pulse carbametry. Such theory was applied to the aforementioned measurements to conclude whether the slight variations observed between HHb and ${\mathrm{CO}}_{2}\mathrm{Hb}$ absorption spectra could be used in such context. Our observations show that such approach seems extremely challenging since these spectra are almost identical. In particular, on the basis of current knowledge, pulse carbametry may not be used in medical practice.

Yet, this work may benefit from further investigations to consolidate its conclusions. In particular, only two wavelengths were considered for pulse carbametry. It would be interesting to use more sophisticated multiwavelengths approaches since they usually give better results in the case of pulse oximetry,^20^ although the question of wavelengths selection becomes more complex.^55^^,^^56^
